# Capsaicin as an amphipathic modulator of Na_V_1.5 mechanosensitivity

**DOI:** 10.1080/19336950.2022.2026015

**Published:** 2022-04-12

**Authors:** Luke M. Cowan, Peter R. Strege, Radda Rusinova, Olaf S. Andersen, Gianrico Farrugia, Arthur Beyder

**Affiliations:** a Division of Gastroenterology and Hepatology, Enteric Neuroscience Program (ENSP), Mayo Clinic, Rochester, MN, USA; b Department of Physiology and Biomedical Engineering, Mayo Clinic, Rochester, Mn, USA; c Department of Physiology and Biophysics, Weill Cornell Medical College, New York, NY, USA

**Keywords:** Amphipathic, arrhythmia, capsaicin, electrophysiology, functional gastrointestinal disorder, ion channel, irritable bowel syndrome, mechanosensitivity, mechanotransduction, voltage-gated sodium channel type 5

## Abstract

*SCN5A*-encoded Na_V_1.5 is a voltage-gated Na^+^ channel that drives the electrical excitability of cardiac myocytes and contributes to slow waves of the human gastrointestinal smooth muscle cells. Na_V_1.5 is mechanosensitive: mechanical force modulates several facets of Na_V_1.5’s voltage-gated function, and some Na_V_1.5 channelopathies are associated with abnormal Na_V_1.5 mechanosensitivity (MS). A class of membrane-active drugs, known as amphiphiles, therapeutically target Na_V_1.5’s voltage-gated function and produce off-target effects including alteration of MS. Amphiphiles may provide a novel option for therapeutic modulation of Na_V_1.5’s mechanosensitive operation. To more selectively target Na_V_1.5 MS, we searched for a membrane-partitioning amphipathic agent that would inhibit MS with minimal closed-state inhibition of voltage-gated currents. Among the amphiphiles tested, we selected capsaicin for further study. We used two methods to assess the effects of capsaicin on Na_V_1.5 MS: (1) membrane suction in cell-attached macroscopic patches and (2) fluid shear stress on whole cells. We tested the effect of capsaicin on Na_V_1.5 MS by examining macro-patch and whole-cell Na^+^ current parameters with and without force. Capsaicin abolished the pressure- and shear-mediated peak current increase and acceleration; and the mechanosensitive shifts in the voltage-dependence of activation (shear) and inactivation (pressure and shear). Exploring the recovery from inactivation and use-dependent entry into inactivation, we found divergent stimulus-dependent effects that could potentiate or mitigate the effect of capsaicin, suggesting that mechanical stimuli may differentially modulate Na_V_1.5 MS. We conclude that selective modulation of Na_V_1.5 MS makes capsaicin a promising candidate for therapeutic interventions targeting MS.

## Introduction

The *SCN5A*-encoded voltage-gated sodium channel, Na_V_1.5, is an ion channel gated by the electrical transmembrane potential present in excitable cells [1–4]. In addition to being voltage-gated, Na_V_1.5 is also mechanosensitive: mechanical force modulates Na_V_1.5's voltage-dependent operation [5,6]. This mechanosensitivity (MS) contributes to a coupled mechano-electrical feedback mechanism that drives contractile response in the mechanically active tissues of the heart and human gastrointestinal (GI) tract. Na_V_1.5 is responsible for the action potential upstroke in cardiac myocytes [3]; and electrical slow waves in human interstitial cells of Cajal (ICC) and intestinal smooth muscle cells (SMC) [7–9]. Na_V_1.5 channelopathies with abnormal MS are found in human cardiac and GI diseases [10–16]. Some *SCN5A* mutations responsible for cardiac arrhythmias result in impaired stretch modulation [14,17]; other mutations associated with altered MS in Na_V_1.5 are found in patients with irritable bowel syndrome (IBS) [16,18]. Some IBS-associated *SCN5A* mutations have relatively unchanged voltage-dependent gating but a loss of MS [17], suggesting that the two mechanisms may be distinct processes that can be targeted separately. However, channelopathies associated with Na_V_1.5 MS dysfunction are poorly studied, and pharmacological treatments targeting MS remain unexplored [14,18,19].

Ion channels are prime pharmacological targets because they are involved in many diseases and being embedded in the cells’ plasma membrane—are highly accessible [20,21]. In patch-clamp studies, mechanical stimuli modulate Na_V_1.5's voltage-dependent function by increasing whole-cell conductance, shifting the voltage-dependence to hyperpolarized potentials, and accelerating kinetics [5,22]. Na_V_1.5 and other mechanosensitive channels detect mechanical stimuli through lipid-bilayer (membrane) tension and cytoskeletal deformation [23–25]. A class of membrane-active drugs, known as amphiphiles, have unique MS modulating properties and are frequently used to alter channel function [21,26,27]. The principal mechanism of action with amphiphiles may be associated with a lipid-bilayer modulation mechanism [28].

Interestingly, some amphipathic drugs modulate both Na_V_1.5's voltage-dependent function and MS [21,26,29]. Ranolazine is a piperazine derivative and a therapeutic amphipath used in the treatment of chronic angina [30]. Ranolazine inhibits Na_V_1.5 late current and MS in primary human GI SMCs, in addition to stretch-dependent function in GI smooth muscle [21,26]. These modulations may contribute to therapeutic outcomes [27] and help explain commonly reported side effects [7,30]. Other amphiphiles frequently used to treat cardiac conditions, such as the antiarrhythmic, amiodarone [31]; and the β-blocker, propranolol [32]; also alter GI motility. The effects observed with these and other amphiphiles relate to their ability to modulate the voltage-gated activation and MS of Na_V_1.5 and other channels.

Inhibiting Na_V_1.5's voltage-dependent opening is usually undesired. Yet, selectively targeting Na_V_1.5 MS while sparing voltage-gated activation could have novel therapeutic applications: this would allow mechano-electrical feedback modulation without direct inhibition of electrical activity. Amphiphilic drugs, including some antiarrhythmics, alter the membrane bilayer within the therapeutic range, and their efficacy has been correlated with membrane-modifying capacity [28,33]. Nonspecific membrane modulation may produce desirable effects, including changes in MS seen with some amphiphiles [28]. Therefore, we screened amongst membrane modifying and therapeutic amphiphiles for an amphipathic agent with minimal inhibition of Na_V_1.5's voltage activation. Among the candidates, capsaicin shows promise; accordingly, we characterized its ability to modulate Na_V_1.5 MS.

## Methods

### Heterologous expression and cell culture

We used the wild-type *SCN5A* variant, Q1077del Na_V_1.5 [1], which makes up 65% of the mRNA transcripts for Na_V_1.5 in the heart [1]. *SCN5A* was co-transfected with pEGFP-C1 into HEK-293 cells using Lipofectamine 3000 (Thermo Fisher Scientific, Massachusetts, USA).

### Amphiphilic drugs

The amphiphiles chosen were readily partitionable based on the octanol-water partition coefficients (logP_ow_): amiodarone [34] (7.2 µM), propranolol (3.48 µM) [35], Triton X-100 [36] (4.6 µM) and Capsaicin [37] (3.04 µM). Amiodarone [31] and propranolol [38] are amphiphiles with known therapeutic potential. Amiodarone and propranolol see common use for antiarrhythmic and antihypertensive effects, respectively. Though most proposed effects involve calcium channels for these amphiphiles, these agents are capable of modifying the membrane bilayer as measured by the gramicidin channel (gA) channel assay to exert off target effects that may include modulation of MS in Na_V_’s [28]. Comparably, capsaicin and Triton X-100 demonstrated membrane modifying potential [39–42].

### Electrophysiology

#### Pipette fabrication

For whole-cell experiments, electrodes were pulled on a P-97 puller (Sutter Instruments, CA) from KG12 glass to a resistance of 2–5 MΩ. For cell-attached patch experiments, electrodes were pulled from 8250 glass (King Precision Glass, California, USA) then fire-polished to wide-bore, bullet-shaped tips with a final resistance of 1–2 MΩ. Electrodes were coated with R6101 elastomer (Dow Corning, MI) and then cured with a heat gun to reduce capacitive transients.

#### Data acquisition

Whole-cell and cell-attached patch data from HEK-293 cells were recorded at 20 kHz with an Axopatch 200B patch-clamp amplifier, Digidata 1550, and pClamp11 software (Molecular Devices, CA).

#### Cell-attached patch

##### Solutions

The pipette solution contained (in mM): 149 NaCl, 4.7 KCl, 2.5 CaCl_2_, 10 HEPES, and 5.5 glucose; with an osmolality of 290 mmol/kg. GdCl_3_ (10 µM) was included in the pipette solution to inhibit endogenous stretch-activated channels [39]. The bath solution contained (in mM): 139 CsCl, 15 NaCl, 4.7 KCl, 2.5 CaCl_2_, 10 HEPES, and 5.5 glucose; with an osmolality of 305 mmol/kg. Both solutions were made to a pH of 7.35. Where applicable, capsaicin was diluted 1000-fold in bath solution from a 20 mM ethanol stock then added to the recording chamber. Seal pressures were digitally controlled and monitored by High-Speed Pressure Clamp (HSPC-2, ALA Scientific, NY). Suction ≤10 mmHg was applied to establish giga-seals.

##### Episodic protocol and mechanical stimulation by pressure

Na^+^ currents in macroscopic patches were elicited by an identical pair of voltage ladders with 31-ms pressure steps up to −50 mmHg encompassing the second voltage ladder. Patches were held at +100 mV, stepped briefly for 10 ms to +190 mV to close Na_V_ channels, then stepped through a 10-step voltage ladder from +100 to 0 mV in 21-ms long, 10-mV increments with a total duration of 280 ms per sweep. Recordings were an average of 5 runs. Capsaicin (20 µM) was added to the chamber 5 min before testing the effects of the drug.

##### Recovery from inactivation

To test the effect of pressure on the recovery of Na_V_1.5 from inactivation, cells were held at 120 mV and stepped to (1) 20 mV for 30 ms, next to (2) 120 mV for a variable duration to recover, then to (2) 20 mV for 30 ms. The time between the beginning of each sweep was 5 s. The duration of the recovery time in stage (2) was varied between 1 and 300 ms in half-log unit increments. The pressure step per sweep was 400 ms regardless of recovery time.

##### Use-dependent inactivation

To test the effect of pressure on the onset (use dependence) of Na_V_1.5 inactivation, cells were held at 120 mV and stepped 20 times to 20 mV, with the frequency between steps varying between 33.33 and 3.33 Hz. The duration of the pressure step per sweep was 30 ms.

#### Whole-cell voltage clamp

##### Solutions

The intracellular solution contained (in mM): 125 CsCH_3_SO_3_, 20 CsCl, 5 NaCl, 5 MgCl_2_, 10 HEPES, and 2 EGTA; with an osmolality of 290 mmol/kg; and pH of 7.0. The extracellular solution contained (in mM): 140 CsCl, 15 NaCl, 5 KCl, 2.5 CaCl_2_, 10 HEPES and 5.5 glucose; with an osmolality of 300 mmol/kg; and pH of 7.35.

##### Peak current, voltage dependence of activation, and kinetics of activation and inactivation

**Figure 1.**
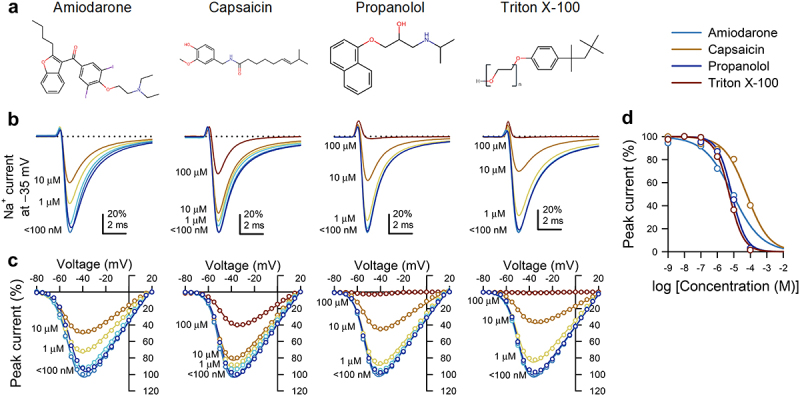
Amphipathic compounds inhibit voltage-gated Na^+^ currents from Na_V_1.5 channels expressed in HEK293 cells. (*a*), Molecular structures of the amphipaths (from *left* to *right*): amiodarone, capsaicin, propranolol, and Triton X-100. (b*-c*), Representative Na^+^ currents elicited by a step from −120 to the −35-mV test voltage (*b*), and peak Na^+^ current-voltage plots across all test voltages (*c*) with 10^−9^ to 10^−^[4] M (*blue-red spectrum*) of membrane-permeable amphipathic compounds in the extracellular solution. (*d*), Dose-response curves for maximum peak Na^+^ current of Na_V_1.5 *vs*. amphipathic concentration; IC_50_ values: amiodarone, 8.4 µM; capsaicin, 60.2 µM; propranolol, 7.6 µM; Triton X-100, 5.3 µM.

**Figure 2.**
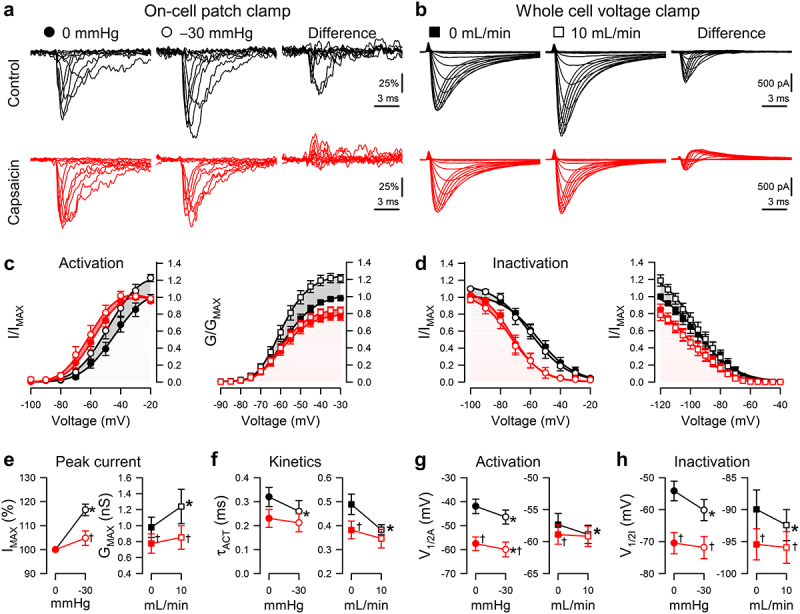
Capsaicin inhibits pressure- and shear-sensitivity of Na_V_1.5. (*a*), Representative Na_V_1.5 currents elicited by voltage ladders ranging −100 to 0 mV in a cell-attached patch (*a*) or −120 mV to −30 mV in a whole cell (*b*), recorded at rest (*filled symbols*) or with force (*empty symbols*), in the presence of 0 µM (*black*) or 20 µM capsaicin (*red*). Difference currents were constructed by subtracting the control Na^+^ currents from the pressure- (*a*) or shear-stimulated (*b*) currents. (c*-d*), Steady-state activation (*c*) and inactivation (*d*) curves of Na^+^ currents in cell-attached patches (*left*) or whole cells (*right*), recorded at rest (*filled symbols*) or with force (*empty symbols*), in the presence of 0 µM (*black*) or 20 µM capsaicin (*red*). (e*-h*), Maximum peak Na^+^ current (*e*), time constant of activation (*f*), and voltage dependence of activation (*g*, V_1/2A_) or inactivation (*h*, V_1/2I_), recorded with 0 or −30 mmHg pressure in the patch (*left*) and 0 or 10 mL/min flow rate in whole cells (*right*) in the presence of 0 µM (*black*) or 20 µM capsaicin (*red*). n = 12–24 cells, *P < 0.05 comparing 0 to −30 mmHg or 0 to 10 mL/min, †*P* < 0.05 comparing 0 to 20 µM capsaicin by a 2-way ANOVA with Tukey posttest.

**Figure 3.**
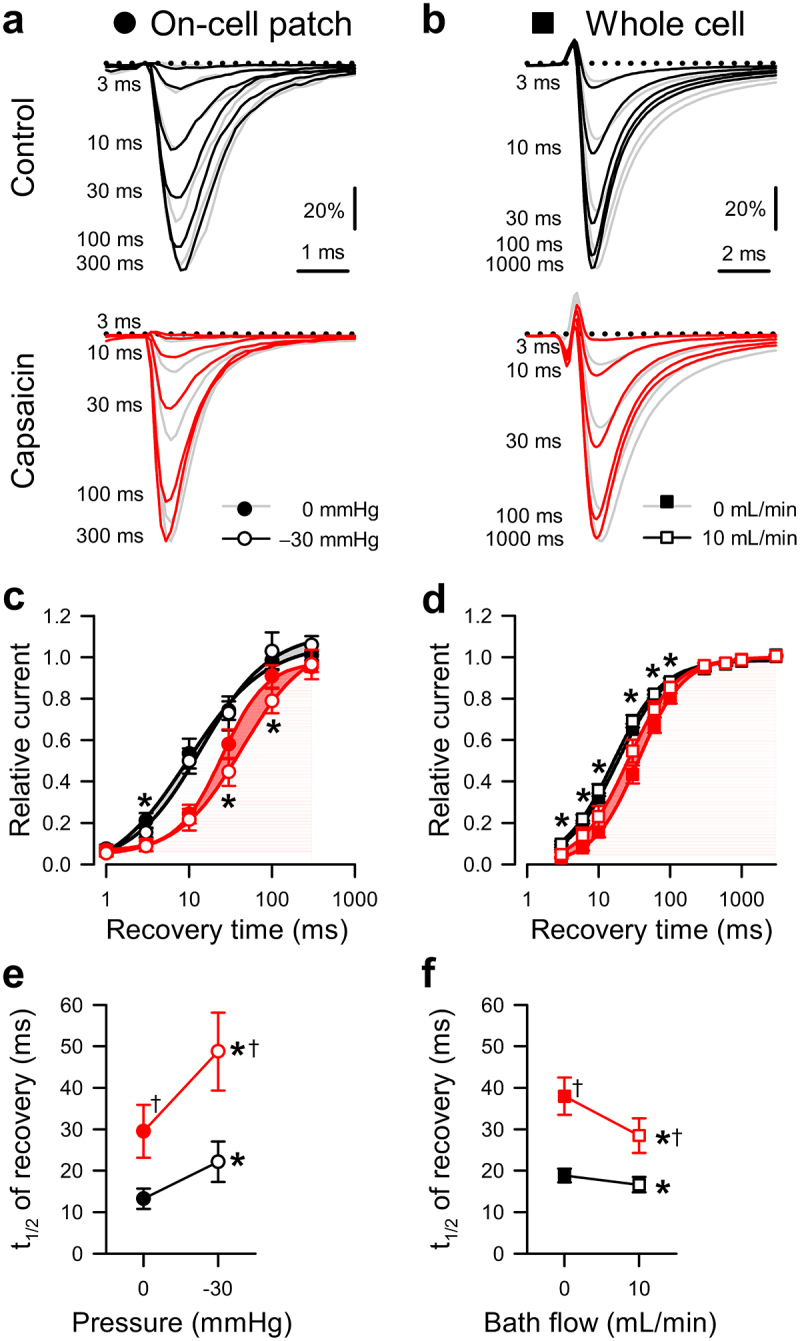
Effects of capsaicin on mechanosensitivity of Na_V_1.5 inactivation recovery time. (a*-b)*, Representative Na_V_1.5 currents at −20 mV in a cell-attached patch (*a*, ●) or −30 mV in a whole cell (*b*, ■), elicited after recovering from the control step for 3–300 ms at −120 mV (*a*) or 3–1000 ms at −130 mV (*b*). Na^+^ currents were recorded at rest (*gray*) or with force (*black and red traces: a*, −30 mmHg pressure; *b*, 10 mL/min shear stress) in the presence of 0 µM (*top*) or 20 µM capsaicin (*bottom*). (c*-d*), Normalized peak Na^+^ current versus recovery time in the presence of 0 µM (*black*) or 20 µM capsaicin (*red*), at 0 (●) or −30 mmHg pressure (○) in the patch (*c*) or at 0 (■) or 10 mL/min (□) shear stress in whole cells (*d*). (e*-f*), Inactivation recovery times (t_1/2_) versus 0 or −30 mmHg pressure in the patch (*e*) and 0 or 10 mL/min shear stress in whole cells (*f*) with 0 µM (*black*) or 20 µM capsaicin (*red*). n = 8–11 cells, *P < 0.05 comparing 0 to −30 mmHg or 0 to 10 mL/min, †*P* < 0.05 comparing 0 to 20 µM capsaicin by a 2-way ANOVA with Tukey posttest.

**Figure 4.**
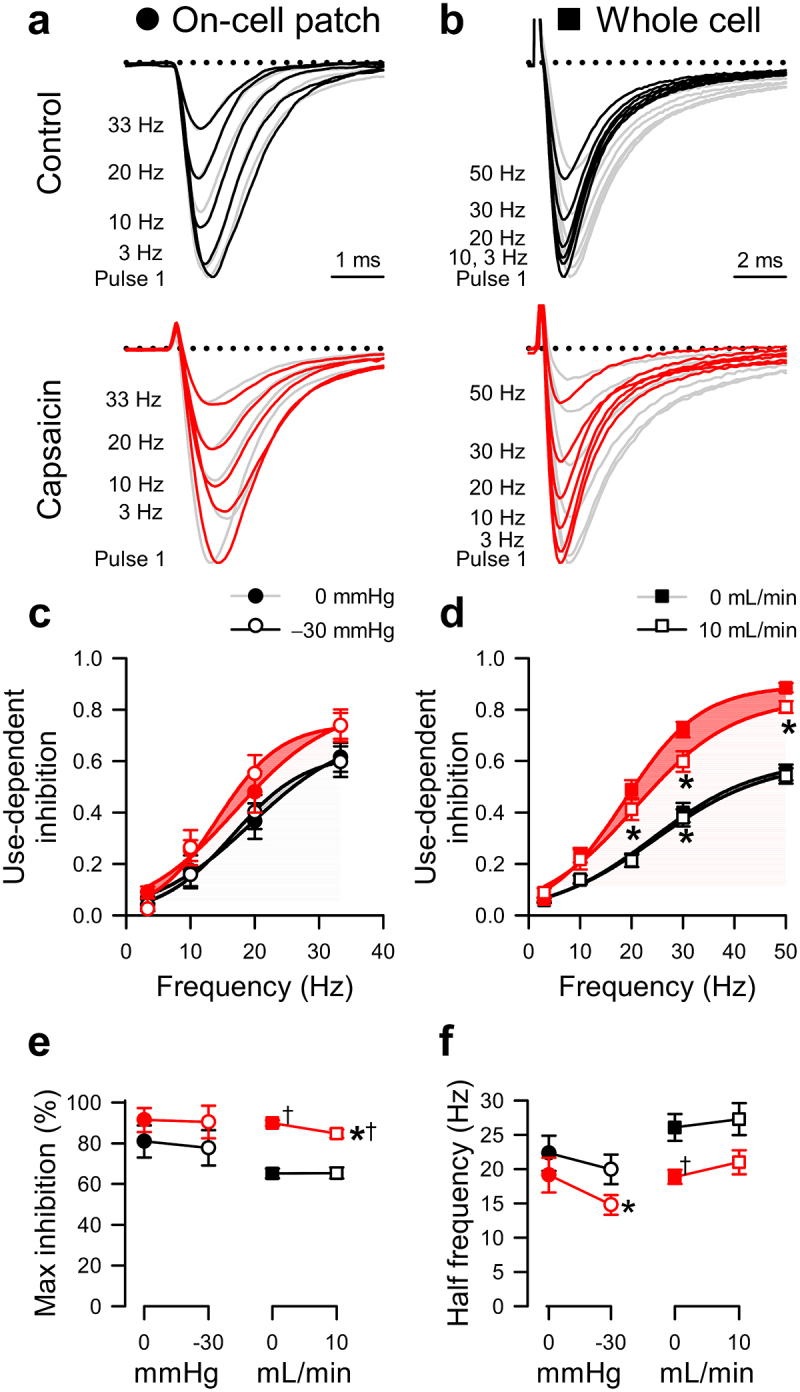
Effects of capsaicin on mechanosensitivity of Na_V_1.5 use-dependent inactivation. (*a-b)*, Representative Na_V_1.5 currents at the 20^th^ step to −20 mV in a cell-attached patch (*A*, **·**) or to −40 mV in a whole cell (*b*, ■), elicited at intersweep frequencies 3–33 Hz (*a*) or 3–50 Hz (*b*). Na^+^ currents were recorded at rest (*gray*) or with force (*black and red traces: a*, −30 mmHg pressure; *b*, 10 mL/min shear stress) in the presence of 0 µM (*top*) or 20 µM capsaicin (*bottom*). (c*-d*), Use-dependent inhibition of peak Na^+^ current versus intersweep frequency in the presence of 0 µM (*black*) or 20 µM capsaicin (*red*), at 0 (●) or −30 mmHg pressure (○) in the patch (*c*) or at 0 (■) or 10 mL/min (□) shear stress in whole cells (*d*). (e*-f*), Maximum use-dependent inhibition (*e*) or frequency of use-dependent inhibition (*f*) versus pressure in the patch (*left*) and shear stress in whole cells (*right*) with 0 µM (*black*) or 20 µM capsaicin (*red*). n = 8–18 cells, *P < 0.05 comparing 0 to −30 mmHg or 0 to 10 mL/min, †*P* < 0.05 comparing 0 to 20 µM capsaicin by a 2-way ANOVA with Tukey posttest.

To measure peak Na^+^ current density, cells transfected with Na_V_1.5 were held at −120 mV then stepped through a 2-stage, 19-step voltage ladder (1) from −110 to −30 mV in 5 mV intervals for 2.9 s each and (2) to −30 mV for 100 ms. The time from the start of each sweep to the next was 5 s. Peak currents at each voltage step were normalized to the cell capacitance (pF) to quantify current densities (Figure 1 and 2) or to the maximum peak inward current without shear to quantify the change in current over baseline (Figures 3–4).

##### Recovery from inactivation

Recovery from inactivation was measured by holding cells at −130 mV and stepping through a 3-stage, 10-step protocol to (1) −30 mV for 100 ms, next to (2) −130 mV for a variable duration to recover, then to (2) −30 mV for 100 ms. The time between each sweep start was 2.5 s. The length of the recovery time in stage (2) of sweep *n* was 4*2 *^n^* ms for a total of *n* = 10 sweeps.

##### Use-dependent inactivation

To measure the onset of Na_V_1.5 inactivation, cells were held at −130 mV and depolarized 10 times to −40 mV, in which the frequency of steps recorded ranged between 0.3 and 50 Hz. *Mechanical stimulation by shear stress*. When testing the effect of shear stress, the extracellular (bath) solution was perfused by gravity drip (at 10 mL/min) for the duration of the voltage protocol.

### Data analysis

The maximum peak Na^+^ current and voltage dependence of activation were determined by fitting the Na_V_1.5 current-voltage (I–V) plots with I = G_MAX_*(V-E_REV_)/(1 + e^(V–V1/2A)/δV^), where *G_MAX_* is the maximum Na^+^ conductance in whole cells (*I_MAX_* substitutes *G_MAX_* for the maximum Na^+^ current in patches), *V* is the voltage, *E_REV_* is the reversal potential, *V_1/2A_* is the voltage of half-maximal activation, and *δV* is the slope. Conductance measurements were performed for the whole-cell configuration. Activation kinetics were determined by fitting currents with a two-term weighted exponential function: *I(t) = A_1_e^(-t/τA)^+A_2_e^(-t/τI)^+C*, where *τ_A_* and *τ_I_* are the time constants of activation and inactivation, respectively, and *A_1_,A_2_*, and *C* are constants. Steady-state inactivation was obtained by fitting remaining peak Na^+^ currents with a 3-parameter sigmoid curve: *I = 1/(1 + e^((V–V1/2I)/δVI)^)*, where *V* is the voltage, *V_1/2I_* is the half-point of steady-state inactivation (availability), and *δV_I_* the slope. For graphing, each IV curve was normalized to the peak effect without mechanical stimulation to demonstrate the increases in peak current with mechanical stimulation. To calculate recovery from inactivation, peak Na^+^ currents were fit with the equation: *I/I_0_ = 1/(1 + t/t_1/2_)^b^*, in which *I/I_0_* is the ratio of Na^+^ current recovered following inactivation from the control current, *b* is the rate of inactivation recovery, *t* is time, and *t_1/2_* is the midpoint in which half of the Na^+^ current has recovered from inactivation. To calculate the plateau for use-dependent inactivation, peak Na^+^ currents of successive pulses were fit with the 3-parameter exponential decay equation: *I_10_/I_1_ = I_ƒ_e^b/(t+c)^*, in which *I_10_/I_1_* is the peak Na^+^ current of step 10 normalized to the peak of step 1, and *I_ƒ_* is the maximally inactivated peak Na^+^ current at frequency *ƒ, t* is time, and *b* or *c* is the rate or constant of use-dependent inhibition, respectively. To determine the voltage-step frequency at which peak Na^+^ currents were inhibited by 50% (or half-frequency of use-dependent inhibition), *I_ƒ_* was plotted *vs*. step frequency *ƒ* and fit with *I_ƒ_ = (1-a)/(1 + e^(ƒ1/2-ƒ)/δV^)*, where *a* is the limit of use-dependent inhibition, *ƒ_1/2_* the half-frequency of use-dependent inhibition, *ƒ* the frequency and *δV* the slope. Data are expressed as the mean ± standard error of the mean (SEM). Significance was assigned when *P* < 0.05 by a 2-way ANOVA and Tukey posttest when comparing force to rest or capsaicin to drug-free.

## Results

### The screen of amphipathic membrane-permeable drugs

As a preliminary screen, we examined select membrane-bilayer-modifying amphipathic agents with high partition coefficients (Table 1) as potential modulators of Na_V_1.5 MS [34–38]. We selected amphiphiles with known therapeutic potential and membrane stiffness modifying properties as previously examined with a gA channel assay [31,38,40–42]. Each compound was tested (10^−9^ to 10^−4^ M) for its ability to inhibit peak voltage-gated Na^+^ currents (Figure 1(a,d)). Triton X-100 was the most potent (log*P*_OW_ 4.6, IC_50_ 5.3 µM, slope 1.00; Figure 1(c,d), Table 1) and capsaicin the least potent (log*P*_OW_ 3.04, IC_50_ 60.2 µM, slope 0.64; Figure 1(c,d), Table 1). The antiarrhythmic amiodarone (log*P*_OW_ 7.2, IC_50_ 8.4 µM, slope 0.50; Figure 1(c,d)) and β-blocker propranolol (log*P*_OW_ 3.48, IC_50_ 7.6 µM, slope 1.01; Figure 1(c,d)) also inhibited Na_V_1.5. Propranolol, which had a partition coefficient (log*P*_OW_) similar to capsaicin, was an 8-fold more potent Na_V_1.5 inhibitor than the latter, indicating that log*P*_OW_ is not a good predictor of the drug’s effect on Na_V_1.5 voltage-gated function, consistent with the previous literature [43]. Similarly, we did not observe a discernable trend in the Hill slope for inhibition – amiodarone and capsaicin had the lowest Hill slopes (0.50 and 0.64, respectively). Nevertheless, amiodarone’s potency (IC_50_ 8.4 µM) was comparable to the two most potent current inhibitors: Triton X-100 and propranolol (IC_50_ 5.3 and 7.6 µM, respectively). Overall, capsaicin, compared against the other amphiphiles tested, was an order of magnitude less potent for current inhibition (IC_50_ 60.2 µM for capsaicin vs. IC_50_ 5.3 to 8.4 µM for the other amphiphiles tested; Figure 1(c,d), Table 1). Because our goal was to find a candidate that would selectively modulate MS while minimizing Na_V_1.5 voltage-dependent current inhibition, we chose capsaicin (20 µM) for further investigation, as this dose inhibited voltage-dependent Na^+^ current by ≤25% (Figure 1(b,d)).

**Table 1.** Partition coefficients and IC_50_ values for amphipathic agents. Partition coefficients denoted log*P*_OW_ for amiodarone, capsaicin, propranolol, and Triton-X100 were previously reported [34–37]. IC_50_, concentration at which an amphipathic agent inhibited half of the maximum peak whole cell Na^+^ current from HEK293 cells transfected with Na_V_1.5. Slope, the hill slope to mechanistically characterize drug behavior based on the slope of fit

	Partition coefficient (log*P*_OW_)	IC_50_ (µM)	Slope
Amiodarone	7.2	8.4	0.50
Capsaicin	3.04	60	0.64
Propranolol	3.48	7.6	1.01
Triton X-100	4.6	5.3	1.00

### Capsaicin inhibits increases in peak current and acceleration with mechanical stimuli

To test the effect of capsaicin on Na_V_1.5 MS, we used two established complementary approaches for mechanical stimulation [44]: (1) the cell-attached macroscopic patch with suction and (2) the whole-cell configuration with fluid shear stress (Tables 2–3, Figure 2). These complementary techniques allow us to validate the parameters of channel MS [17,44–47]. The pressure effect was tested in a pairwise fashion [17,26,48], with pressure at 0 or −30 mmHg applied at each voltage step (Figure 2(a-h)). Whole-cell current response to shear was tested by perfusion at 0 or 10 mL/min (Figure 2b, d, e-h). We then reassessed the function in both configurations in the presence of 20 µM capsaicin (Tables 2–3, Figure 2(a-h)). Suction increased normalized peak currents (I_MAX_) by 16.6 ± 2.4% (*P* < 0.05; n = 24; Figure 2(a-e)), and shear increased the peak current (I_PEAK_) by 16.0 ± 3.1% in whole cells (0.26 ± 0.10 nS increase in conductance; *P* < 0.05, 0 to 10 mL/min; n = 12; Figure 2(b-e)). Capsaicin decreased I_PEAK_ by 22.1 ± 3.9% (*P* < 0.05, 0 to 20 µM capsaicin), and both pressure (+4.8 ± 3.0%) and shear sensitivity (+3.1 ± 3.8%, +0.08 ± 0.05 nS) were lost (n = 12–14; *P* > 0.05 to drug with no force).

**Table 2.** Effect of capsaicin on pressure-induced Na_V_1.5 mechanosensitivity in cell-attached patches. Effects of pressure (0 or −30 mmHg) on parameters of macroscopic Na^+^ currents without (0 µM) or with capsaicin (20 µM): maximum peak Na^+^ currents normalized to controls at 0 mmHg (I_MAX_), voltage dependence of activation (V_1/2A_) or inactivation (V_1/2I_), time constant of activation (τ_A_), time of inactivation recovery (t_1/2R_), slope of inactivation recovery (slope), maximum use-dependent inhibition (block), frequency of use-dependent inhibition (ƒ_1/2_). n = 8–24 cells, *P < 0.05, 0 to −30 mmHg or †*P* < 0.05, 0 to 20 µM capsaicin by a 2-way ANOVA with Tukey posttest

	0 mmHg	0 µM -30 mmHg	Change	n	0 mmHg	20 µM -30 mmHg	Change	n
I_MAX_ (%)	100.0 ± 0.0	116.6 ± 2.4*	16.6 ± 2.4	24	100.0 ± 0.0	104.8 ± 3.0^†^	4.8 ± 3.0	14
V_1/2A_ (mV)	−41.9 ± 3.0	−46.4 ± 3.0*	−4.5 ± 0.6	24	−57.5 ± 2.8^†^	−60.0 ± 3.1*^†^	−2.4 ± 0.6	14
V_1/2I_ (mV)	−54.1 ± 3.1	−60.1 ± 3.3*	−6.0 ± 0.9	24	−70.5 ± 3.3^†^	−71.9 ± 3.5^†^	−1.4 ± 1.2	14
τ_A_ (ms)	0.32 ± 0.04	0.26 ± 0.04*	−20.0 ± 5.3%	24	0.23 ± 0.04	0.21 ± 0.04	−11.0 ± 5.4%	14
t_1/2R_ (ms)	13.2 ± 2.5	22.1 ± 4.9*	8.9 ± 3.9	11	29.5 ± 6.4^†^	48.7 ± 9.4*^†^	19.2 ± 8.6	11
Slope (ms^−^[^1]^)	1.3 ± 0.1	1.2 ± 0.1	−0.10 ± 0.17	11	1.9 ± 0.3	2.3 ± 0.8	0.41 ± 0.87	11
Block (%)	80.9 ± 7.9	77.8 ± 8.6	3.2 ± 9.4	11	93.6 ± 4.5	85.5 ± 8.5	4.7 ± 3.8	8
ƒ_1/2_ (Hz)	22.3 ± 2.6	20.0 ± 2.1	−2.4 ± 3.3	11	19.1 ± 2.5	14.8 ± 1.4*	−4.2 ± 1.4	8

**Table 3.** Effect of capsaicin on shear-induced Na_V_1.5 mechanosensitivity in whole cells. Effects of shear stress (0 or 10 mL/min) on parameters of whole cell Na^+^ currents without (0 µM) or with capsaicin (20 µM): maximum peak conductance (G_MAX_), maximum peak current density (I_PEAK_), voltage dependence of activation (V_1/2A_) or inactivation (V_1/2I_), time constants of activation (τ_A_) and fast (τ_F_) or slow inactivation (τ_S_), time of inactivation recovery (t_1/2R_), slope of inactivation recovery (slope), maximum use-dependent inhibition (block), frequency of use-dependent inhibition (ƒ_1/2_). n = 8–18 cells, *P < 0.05, 0 to 10 mL/min or †*P* < 0.05, 0 to 20 µM capsaicin by a 2-way ANOVA with Tukey posttest

	0 mL/min	0 µM 10 mL/min	Change	n	0 mL/min	20 µM 10 mL/min	Change	n
G_MAX_ (nS)	0.98 ± 0.13	1.24 ± 0.22*	0.26 ± 0.10	12	0.78 ± 0.12^†^	0.85 ± 0.15^†^	0.08 ± 0.05	12
I_PEAK_ (pA/pF)	−66.8 ± 12.3	−86.6 ± 17.7*	16.0 ± 3.1%	12	−56.7 ± 58.5^†^	−58.5 ± 11.6^†^	3.1 ± 3.8%^†^	12
V_1/2A_ (mV)	−57.3 ± 1.7	−58.9 ± 1.4*	−1.5 ± 0.6	12	−58.9 ± 1.5^†^	−59.2 ± 1.6	−0.3 ± 0.1	12
V_1/2I_ (mV)	−90.3 ± 3.3	−92.7 ± 2.6*	−2.5 ± 0.9	12	−95.4 ± 2.6^†^	−95.8 ± 2.7^†^	−0.5 ± 0.6	12
τ_A_ (ms)	0.5 ± 0.05	0.39 ± 0.02*	−20.4 ± 3.3%	12	0.39 ± 0.04^†^	0.38 ± 0.04	−1.3 ± 7.0%^†^	12
τ_F_ (ms)	0.82 ± 0.08	0.57 ± 0.04*	−27.5 ± 4.0%	12	0.80 ± 0.08	0.56 ± 0.05*	−25.1 ± 5.9%	12
τ_S_ (ms)	4.7 ± 0.3	3.5 ± 0.2*	−24.2 ± 4.4%	12	3.8 ± 0.3^†^	2.9 ± 0.2*^†^	−20.5 ± 4.8%	12
t_1/2R_ (ms)	18.8 ± 1.7	16.6 ± 1.8*	−2.2 ± 0.6	8	38.0 ± 4.5^†^	28.4 ± 4.2*^†^	−9.5 ± 0.9^†^	8
slope (ms^−^[^1]^)	−1.26 ± 0.04	−1.28 ± 0.05	−0.02 ± 0.02	8	−1.43 ± 0.02^†^	−1.42 ± 0.06	0.01 ± 0.04	8
Block (%)	64.6 ± 2.4	64.7 ± 2.6	0.2 ± 1.7	18	89.0 ± 1.4^†^	83.9 ± 2.0*^†^	−5.1 ± 0.9^†^	10
ƒ_1/2_ (Hz)	26.1 ± 2.0	27.3 ± 2.3	1.2 ± 0.9	18	18.9 ± 1.0^†^	21.0 ± 1.7	2.1 ± 1.4	10

In the absence of drug, mechanical force accelerated Na^+^ current activation, decreasing the activation constant (τ_ACT_) by 20.0 ± 5.3% or 20.4 ± 3.3%, respectively (n = 12–14; *P* < 0.05 to no force controls; Figure 2(f)). Capsaicin accelerated Na_V_1.5 activation by 20.3 ± 6.9% at rest (n = 12–14; *P* < 0.05, 0 to 20 µM capsaicin) in whole cells but not in patches, and it inhibited the acceleration of activation induced by pressure and shear, as τ_ACT_ did not accelerate with pressure or shear (−11.0 ± 5.4% or −1.3 ± 7.0%, respectively; n = 12–14; *P* > 0.05 to drug with no force; Figure 2(f)). In all, capsaicin inhibits Na_V_1.5's mechanosensitive increases in peak current and accelerations in kinetics.

### Capsaicin inhibits mechanically induced hyperpolarizing shifts in the voltage dependence of activation and channel availability

Pressure [6,21,26,48], and shear [17,18,25] produce hyperpolarizing shifts in the voltage dependence of Na_V_1.5 activation and inactivation. Membrane-permeable amphipathic drugs like lidocaine and ranolazine reduce these mechanosensitive shifts in voltage dependence [21,26]. Therefore, we explored whether capsaicin could reduce the pressure- or shear-induced shifts in voltage dependence. Comparable to our previous work without drug [21,26], suction (−30 mmHg) produced a leftward shift of −4.5 ± 0.6 mV in the voltage dependence of activation (V_1/2A_), and shear stress induced a smaller but significant shift of −1.5 ± 0.6 mV in the V_1/2A_ (*P* < 0.05 to no force; Tables 2–3, Figure 2(c-g)). Without force, capsaicin produced a hyperpolarizing shift in V_1/2A_ (−1.6 ± 0.4 mV; *P* < 0.05, 0 to 20 µM capsaicin) in whole cells. With force, capsaicin inhibited the shear-induced shift in the V_1/2A_ (−0.3 ± 0.1 mV; *P* > 0.05 to drug with no shear) but not the pressure-induced shift (−2.4 ± 0.6 mV; *P* < 0.05 to drug with no pressure). Similar to shear-induced shifts in the V_1/2A_, pressure or shear shifted the voltage dependence of inactivation or availability (V_1/2I_) in the absence of capsaicin (−6.0 ± 0.9 mV with pressure or −2.5 ± 0.9 mV with shear; *P* < 0.05 to no force), though the technical limitations of holding the voltage to more negative potentials prevented us from reaching the plateau in whole-cell experiments (Tables 2–3, Figure 2(d-h)). Without force, capsaicin produced a hyperpolarizing shift in the whole-cell V_1/2I_ by −5.1 ± 0.7 mV, as previously observed [42]. In the presence of capsaicin, neither pressure nor shear significantly affected the V_1/2I_ (−1.4 ± 1.2 or −0.5 ± 0.6 mV change, respectively; *P* > 0.05 to drug with no force), suggesting loss of the MS of Na_V_1.5 inactivation. Overall, our results show that capsaicin inhibited the mechanosensitive shifts in Na_V_1.5 voltage-gating.

### Effects of capsaicin and mechanical stimuli on recovery from inactivation

Both capsaicin and pressure delay the recovery of Na_V_1.5 from fast inactivation [42,48]. Therefore, we tested whether the presence of capsaicin affected the recovery from fast inactivation (1 to 1000 ms) in the absence or presence of mechanical stimuli (Tables 2–3, Figure 3(a,b)). Without force or drug, Na^+^ currents recovered within ~100 ms in either configuration (Figure 3(c)); the half-time of Na_V_1.5 inactivation recovery (t_1/2R_) at rest was 13.2 ± 2.5 ms in the patch and 18.8 ± 1.7 ms in whole-cell (Tables 2–3, Figure 3(c-f)). In addition, unlike the consistent responses to force regardless of stimulus or configuration described above, we observed consistent differences between the two approaches when assessing recovery. Shear accelerated Na_V_1.5's t_1/2R_ by 2.2 ± 0.6 ms (*P* < 0.05, 0 to 10 mL/min), whereas pressure delayed the t_1/2R_ (+8.9 ± 3.9 ms; *P* < 0.05, 0 to −30 mmHg) (Tables 2–3, Figure 3(c-f)). In whole cells, without force, capsaicin delayed the recovery from inactivation; the t_1/2R_ increased from 18.8 ± 1.7 to 38.0 ± 4.5 ms (*P* < 0.05, 0 to 20 µM capsaicin). With capsaicin present, pressure increased the t_1/2R_ by 19.2 ± 8.2 ms (*P* < 0.05 to drug with no pressure), whereas shear reduced the t_1/2R_ in whole cells by 9.5 ± 0.9 ms (*P* < 0.05 to drug with no shear). In all, the recovery from inactivation was delayed by capsaicin across both approaches. In the presence of capsaicin, pressure further delayed recovery in pressurized patches, but shear accelerated recovery in whole cells.

### Effects of capsaicin and mechanical stimuli on use-dependent inactivation

Capsaicin can stabilize the inactivated state of Na_V_1.5 through use-dependent inhibition [42]. Therefore, we tested whether force could alter the use-dependent inactivation of Na_V_1.5 in the absence of capsaicin and reexamined use-dependent inactivation in the presence of capsaicin (Figure 4(a-f)). To measure the use-dependent inhibition of Na_V_1.5 expressed in HEK cells, Na^+^ currents elicited by steps to either 0 or −30 mV in patches or whole cells were sampled at 3–33 Hz or 0.3–50 Hz, respectively. Without force or drug, the maximum use-dependent inhibition of Na_V_1.5 was 80.9 ± 7.9% with a half-frequency (ƒ_1/2_) of 22.3 ± 2.6 Hz in patches (Table 2, Figure 4(c-f)) and 64.6 ± 2.4% with a ƒ_1/2_ of 26.1 ± 2.0 Hz in whole cells (Table 3, Figure 4(d-f)). In the absence of capsaicin, the use-dependence did not change with either pressure or shear (*P* > 0.05 to no force; Tables 2–3, Figure 4(c-f)). Without shear force, capsaicin increased the maximum use-dependent inhibition of Na_V_1.5 to 89.0 ± 1.4% and decreased ƒ_1/2_ to 18.9 ± 1.0 Hz (*P* < 0.05, 0 to 20 µM capsaicin) in the whole-cell configuration. In the presence of capsaicin, shear produced a modest decrease in the maximum use-dependent inhibition (5.1 ± 0.9%; *P* < 0.05 to drug with no shear), and ƒ_1/2_ was unaffected, suggesting that shear partially reverses the use-dependent inhibition of Na_V_1.5 promoted by capsaicin. In patches, capsaicin affected neither the use-dependent inhibition nor ƒ_1/2_ at rest (*P* > 0.05, 0 to 20 µM capsaicin) but increased the pressure-sensitivity (ƒ_1/2_ decreased by 2.4 ± 3.3 Hz; *P* < 0.05 to drug with no pressure). Together, our results suggest that, though capsaicin enhances use-dependent inhibition, its effect on force-dependent changes to Na_V_1.5 use dependence may be specific to the type of force applied.

## Discussion

The MS of voltage-gated ion channels contributes to a mechano-electrical feedback system that has important implications in organs with primarily mechanical functions [14,48,49]. Examples of such mechanically active organs include the heart and gut, where ion channels contribute to important physiologic functions [14,50]. In the human heart and gut, the voltage-gated sodium channel, Na_V_1.5 serves as a key electrically excitable component in both the cardiac action potential and GI smooth muscle contraction [17,50]. Besides being voltage-gated, Na_V_1.5 is mechanosensitive [37]; this property contributes to mechano-electrical feedback [49]. Membrane-permeable amphiphiles impact Na_V_1.5 MS [7,21,51] and may be capable of modulating this feedback mechanism to exert therapeutic effects [21].

How membrane-permeable amphipathic drugs alter the MS of voltage-gated channels, like Na_V_1.5, and whether they do so by a mechanism separate from voltage-dependent current inhibition remain critical points for understanding amphiphile-mediated effects [21,26,29]. For example, the membrane-permeable amphiphile and local anesthetic—lidocaine—inhibits Na_V_1.5's peak current, and at lower concentrations, inhibits MS [21,26]. Supporting a separate-mechanisms hypothesis for current inhibition and altered mechanosensation by amphiphiles, the anesthetic-binding site mutation, F1760A, eliminates the voltage-dependent inhibition by lidocaine without altering lidocaine’s effect on MS [21]; while the membrane-impermeant lidocaine analog, QX-314, does not affect MS.

Amphiphiles often have significant effects on both voltage-gating and MS [21]. Thus, we searched for a membrane-permeable amphipathic agent that alters Na_V_1.5 MS without significant inhibition of the voltage-gated channel opening. In principle, this would allow selective targeting Na_V_1.5 MS in conditions where it is abnormal. We selected amphiphiles with high partition coefficients and tested each candidate’s inhibition of peak voltage-gated currents in Na_V_1.5. Our choices were motivated by these molecules’ therapeutic use and ability to alter membrane stiffness, as demonstrated with the gA channel assay [31,38,40–42]. Interestingly, the partition coefficients for these compounds do not predict the drug’s effects on Na_V_1.5's voltage-gated function. Our results, interpreted together with earlier studies with membrane-impermeable quaternary ammonium amphiphilic local anesthetics [52,53], suggest that amphiphile-mediated effects require membrane partitioning as a prerequisite for peak current inhibition. However, the level of partitioning estimated using logP_ow_ does not determine the level of Na_V_1.5 current inhibition.

The class of therapeutic amphiphiles, local anesthetics [52] have been shown to interact with the channel pore to exert current inhibition through intracellular access. Local anesthetics exert open-state block through the channel pore via a local anesthetic-binding site that is accessible during opening events. However, local anesthetics exert poor closed-state block [52,54], which may involve a different mechanism that is shared with other amphiphiles. The Hill slopes, ranging from 0.5 to 1 for the chosen amphiphiles, did not predict the closed-state current inhibition level, suggesting that amphiphile-mediated peak current inhibition does not have a precise binding modality [55]. This may indicate that mechanisms of current inhibition by amphiphiles depend on the identity of the amphiphile used. While propranolol (slope of 1.0) and Triton X-100 (slope of 1.0) could be interacting with a binding site, amiodarone (slope 0.5) and capsaicin (slope 0.6) appear to be acting by another nonspecific mechanism. This finding is supported by earlier work with the gA channel assay and KcsA K^+^ channel. Using the KcsA channel as a model for the voltage-gated ion channel [56], amphiphiles were generally found to alter KcsA function, but amphiphiles altered different gating steps in an identity-specific manner.

Both amiodarone and propranolol are antiarrhythmics, yet amiodarone exerts membrane-perturbing effects within the therapeutic range of use while propranolol-induced perturbations occur above therapeutic concentrations [28]. This may underlie amiodarone’s therapeutic and off-target effects, including changes in MS. In the case of thiazolidinediones, a class of insulin-sensitizing drugs, greater lipid-bilayer altering potency is strongly correlated with efficacy and side effects [33]. This implies that some pharmacological agents alter the membrane to produce desired therapeutic outcomes. Similar to these drugs, capsaicin alters the membrane bilayer at concentrations required to exert effects [42]. These nonspecific membrane-modulatory behaviors may describe one mechanism by which some amphiphiles function therapeutically and alter MS. From our selection however, choosing among amphiphiles with bilayer-modulating effects and minimal closed-state current inhibition as selection criteria may indicate therapeutic potential. Capsaicin inhibited Na_V_1.5 MS in a manner comparable to other amphipaths—such as lidocaine [21,26], and ranolazine [14]—yet with minimal inhibitory effects. Capsaicin consistently inhibited the MS effects of pressure and shear stress on Na_V_1.5 in membrane patches and whole cells, respectively, by inhibiting the: (1) mechanosensitive increases in Na^+^ current, (2) shifts in steady-state voltage-dependence, and (3) acceleration of Na_V_1.5 gating kinetics.

Quantifying ion channel MS is challenging [57], and different approaches often yield different results [23,44]. Few studies have explored Na_V_1.5 MS using both whole-cell and macroscopic patch modes in parallel [17,21,48]. To our knowledge, this is the first study to directly compare pressure and shear effects on the biophysics of Na_V_1.5 MS in the absence or presence of a drug in detail. As with other amphiphiles studied [21,58], capsaicin hyperpolarized parameters of voltage-dependence. In the whole-cell mode, capsaicin has previously demonstrated the ability to shift the V_1/2A_ and V_1/2I_ to hyperpolarized potentials [42]. Comparably, we observed significant shifts in both the patch and whole-cell configuration. Impressively, despite the stimuli being two distinct modes of mechanical stimulation, with unique mechanisms, most of Na_V_1.5's mechanosensitive responses and capsaicin’s effects on Na_V_1.5 MS were similar across techniques. Both produced an increase in peak Na^+^ current, shifts in the V_1/2A_ and V_1/2I_, and an acceleration in τ_A_. Capsaicin in both approaches inhibited most of these changes. These effects are important in the context of MS channel function—mechanical strain leads to faster and greater Na^+^ influx, which increases Na_V_ channel availability and further depolarizes the membrane. When Na_V_1.5 is mechanically stimulated in the presence of capsaicin, there would be a reduction in Na^+^ influx and consequently a slower membrane depolarization, which would reduce the effect of mechanical force on Na_V_ channel availability.

Many membrane modulating amphiphilic drugs alter recovery from inactivation and exert use-dependent block on Na_V_ channels [59–61]: these responses are modulated by capsaicin among other amphiphiles [42]. Changes in channel properties due to mechanical stimuli can be considered mechanosensitive processes [23]. Therefore, it was important to understand how capsaicin could modulate these effects in addition to the commonly studied mechanosensitive parameters of activation and inactivation gating. We found opposite responses in the pressure- and shear-sensitivity of Na_V_1.5 inactivation recovery and use-dependent inactivation. Pressure applied to patches increased the time to recover from inactivation, whereas shear stress applied in the whole-cell configuration decreased it. As previously reported [42], the addition of capsaicin delayed Na_V_1.5's recovery from inactivation in patches and whole cells. Intriguingly, the opposing effects on recovery with mechanical stimuli were further amplified with capsaicin. Capsaicin and pressure cooperatively delayed recovery further, in contrast to whole cells, where shear stress accelerated inactivation recovery, and thus reduced capsaicin mediated recovery delays.

At the concentration we tested, capsaicin alone did not significantly alter the half-frequency for use-dependent inactivation in patches, but it did in whole cells. When capsaicin and pressure were applied together, they decreased the frequency of use-dependent inactivation in patches; while in whole cells, capsaicin alone lowered use-dependence frequency, a process unaffected by shear stress. Pressure has previously been shown to prolong Na_V_1.5 inactivation recovery time in patches [48], but the effect of shear stress on inactivation recovery or use-dependent inhibition of Na_V_1.5 was previously unknown.

The opposing responses in use-dependence and recovery using the two approaches are independent of capsaicin, suggesting that these mechanical stimuli may act through different mechanisms [45,62]. Conceivably, the effect of pressure or shear stress on the membrane or cytoskeleton could be different. Shear can lead to uniaxial elastic tension along the membrane, yielding asymmetrical sliding of lipid membrane leaflets [29,30]. The associated effects of lipid bilayer thinning affect some functional states, such as inactivation, more than others [40,41]. Meanwhile, macroscopic patch suction can create unequal transmembrane surface tension [23,45,63], with most tension at the dome peak. Accordingly, from the perspective of the membrane, two different phenomena may be occurring with each respective stimulus that may translate to divergent effects on Na_V_1.5 MS. Negative pressures in the patch may alter the properties of voltage-gating through a lipid-stretch mechanism, whereas shear stress across the membrane may alter gating though a mechanism involving an asymmetric tension across on the entire cell that is attached to the electrode [23,62,64].

Lipid compositions surrounding channels play critical roles in force transduction and gating mechanisms. Changing the lipid composition of synthetic bilayers alters mechano-gated MscL channel gating parameters—adding lysophosphatidylcholine to vesicles composed of phosphatidylcholine was sufficient to measurably open MscL channels [65]. In another study, charged amphiphiles of a like charge were capable of activating the MscL channel, and effects could be neutralized with the addition of amphiphiles of an opposing charge [66]. Furthermore, the gA channel reporter suggests that membrane elasticity may be essential in the Na_V_ channel gating mechanism [42]. Two gA channel subunits, which function as molecular force transducers, join to form an open pore when the membrane elastic disjoining force is overcome [40]. Amphiphiles like capsaicin and capsazepine increased gA channel appearance rate by lowering the elastic disjoining force energy barrier for gA dimerization [40,42]. These findings suggest the presence of an important membrane-channel force transduction mechanism and that the application of therapeutic amphiphiles may alter/modulate channel behavior by this mechanism. The decrease in membrane stiffness by amphiphiles, such as capsaicin, may be responsible for the loss of mechanosensitive effects observed in our study. Overall, the membrane bilayer likely plays a critical role in channel MS.

Mechanical stimuli modulate components of cardiac and intestinal contractility [24]. In the setting of mechanically active organs, such as the heart and GI, the mechanoelectric feedback loop has important system level regulatory functions [49,67]. This feedback loop could serve as point of therapeutic regulation in the treatment diseases with MS dysfunction [26]. Amphiphiles that blunt mechanosensitive effects, like capsaicin, may reduce MS when mechanoelectric feedback is disrupted in cases of cardiac and gastrointestinal disease [24,68]. Pharmacologic modulation via capsaicin could conceivably reduce channel activity through a combination of use-dependent block and recovery delay, while sparing voltage-gated operation.

Capsaicin joins a growing group of amphipaths that modulate Na_V_1.5 voltage-gating and MS with therapeutic potential. The Na_V_1.5 targeting amphiphilic drug, ranolazine is a common anti-ischemic and anginal medication. Ranolazine inhibits the increase in peak Na^+^ current and the hyperpolarization of voltage-dependence of activation induced by pressure or shear stress in a manner comparable to capsaicin [26]. Abnormalities in gut transit are common side effects of ranolazine [30]. This could be explained by ranolazine’s ability to inhibit both muscle contractility in human colon smooth muscle cells (SMCs), and Na_V_1.5 peak current and MS [7]. Capsaicin activates its canonical target, TRPV1, in sensory neurons to improve GI dysfunction in IBS-D patients [69]; however, TRPV1 is not pressure-sensitive [70] and is minimally expressed in HEK cells [63,71]. Capsaicin has shown promise in targeting IBS pain [69]. Fascinatingly, it also affects gut motility [72–74], possibly through its effects on Na_V_1.5 MS, since TRPV1 is only expressed in extrinsic sensory fibers, which are not primary regulators of motility [75]. Building on this study, there may be a possibility of using capsaicin to affect sensory (TRPV1) and motility (*SCN5A*/Na_V_1.5) processes by different mechanisms in the GI tract. Amphipathic drugs are widely used in clinical practice to target ion channels but are rarely used for mechano-modulation [12,13]. The ability of capsaicin, lidocaine [21], and ranolazine [26] to inhibit Na_V_1.5 MS demonstrates that membrane-partitioning amphipathic agents can effectively alter MS and may have pharmacologic potential. Such amphiphiles may be viable candidates for therapeutic modulation of Na_V_1.5 MS and for targeting dysfunction in channelopathies with disordered MS. Channelopathies involving mechanosensitive dysfunction are an emerging area of study [12,16,17,19,76,77]. Voltage-sensitive mechano-gated Piezo channels [78–80] and Na_V_1.5 MS channelopathies currently lack targeted treatment options. While continued progress is required, this study suggests that therapeutically targeting the voltage-gated and mechanosensitive functions of Na_V_1.5 separately in human diseases may hold promise for MS-associated disorders.
